# Editorial: Polar Genomics in a Changing World

**DOI:** 10.3390/genes14071395

**Published:** 2023-07-03

**Authors:** Joseph Ivan Hoffman, Svenja Heesch, Melody Susan Clark

**Affiliations:** 1 Department of Animal Behavior, University of Bielefeld, Postfach 10 01 31, 33615 Bielefeld, Germany; 2 British Antarctic Survey, High Cross, Madingley Road, Cambridge CB3 OET, UK; 3 Applied Ecology & Phycology, Institute for Biosciences, University of Rostock, 18059 Rostock, Germany

Polar regions play critical roles in the function of the Earth’s climate system, many of which are underpinned by their endemic biota. Whilst being home to some of the world’s best-known charismatic megafauna such as polar bears, whales, penguins, seals and albatrosses, polar regions also harbour some of the most poorly explored and least understood biodiversity on the planet (https://www.ipcc.ch/reports, accessed on 9 June 2023). Moreover, these regions are amongst those areas of our planet experiencing the most rapid rates of warming [1,2], resulting in severe threats to their unique ecosystems [3]. With regional warming, the organisms living in these frozen ecosystems will have to adapt if they are to survive, yet we currently have a very limited understanding of polar biodiversity, or indeed of the future resilience of polar organisms in our changing world. To generate *a priori* predictions of biodiversity change in these regions, it is imperative to understand the true extent of polar biodiversity, including how organisms interact (for example, in food webs), the biological mechanisms by which they have adapted to polar environments, their levels of phenotypic plasticity, and how these attributes may impact their abilities to respond to change. Critical to this understanding are “genomics” approaches that exploit the high-throughput sequencing of genetic material. With the costs of sequencing DNA and RNA having decreased dramatically over recent years, our abilities to probe the genetic code of polar organisms have expanded immeasurably, such that we are now able to answer ecological and evolutionary questions that were intractable even a few years ago, as exemplified by the contributions in this Special Issue on polar genomics.

## 1. Population Genetics, Demographic Histories and Genetic Diversity

Understanding the effects of historical population size changes on the genetic diversity of polar organisms is important for predicting future demographic responses to environmental change. Three papers in this Special Issue applied diverse yet complementary approaches to polar predators to infer genomic footprints of past hunting and habitat loss. Cockerill et al. [4] used whole-genome resequencing to show that climate-change-driven habitat fragmentation is associated with higher levels of inbreeding and increased mutation loads in Arctic foxes from northern Fennoscandia. Their study illustrates how climate change can drive genomic erosion and highlights an important emerging challenge for the conservation management of Arctic carnivores. Hoffman et al. [5] used reduced representation sequencing and site frequency spectrum-based demographic reconstruction to show that competitive prey release due to the hunting of the great whales may have facilitated the demographic recovery of a heavily hunted fur seal population in south Georgia (although the same population is currently declining in response to ongoing environmental change [6,7]). Buss et al. [8] sequenced contemporary and historical mitogenomes to show that southern hemisphere fin whales have retained high levels of genetic diversity despite intensive whaling during the 20th century. Collectively, these studies highlight the importance of understanding species- and context-dependent demographic responses to anthropogenic stressors and their implications for polar ecosystems.

To link patterns of genetic diversity to longer-term processes, Collins et al. [9] surveyed the genetic diversity of four terrestrial mite genera in the Ross Sea region of Antarctica. They uncovered an unexpectedly high diversity of cryptic species, probably reflecting long-term isolation in microhabitats that persisted through numerous glacial cycles. In line with this, Beck et al. [10] reported a relationship between the timing of glaciation and the diversity of lichen-associated eukaryote communities. Using a metabarcoding approach, they found that around 50% more species were present in lichens from sites that have remained deglaciated for at least 5000 years in comparison to more recently deglaciated sites.

## 2. Experimental Approaches to Understand Responses to Environmental Change

While studies of organisms in their natural environments are undoubtedly important for understanding climate change impacts, experimental approaches are needed to evaluate causal relationships and their mechanistic underpinnings. Barrett et al. [11] used gene expression profiling to characterise the stress response of Greenland mussels subjected to increased temperatures and decreased salinities. They found evidence for robust stress responses and considerable resilience to unfavourable environmental conditions. Similarly, Prelle et al. [12] found that six benthic diatom strains from the Antarctic Peninsula exhibited high levels of physiological plasticity and were unexpectedly tolerant to a broad range of temperature and salinity conditions. However, two parallel studies of marine microbial communities reached different conclusions. Ahme et al. [13] incubated water from the Fram straight to +2 °C, +6 °C and +9 °C and used metabarcoding to assess shifts in microbial community composition. They found a marked drop in species richness and phenotypic diversity at the highest temperature, implying a “tipping point” of 6–9 °C for many key Arctic microbial species. A complementary approach was taken by Ilicic et al. [14], who focused on the short-term *in situ* dynamics of marine bacterial communities in response to a naturally occurring temperature anomaly. They used metagenomics to show that short-term warming and increased glacial meltwater run-off had a strong forcing effect at the species level but also appeared to create new niches for opportunistic, faster-growing strains. These and other studies suggest that climate change will likely produce both “winners” and “losers”, with the net effect likely being an overall loss of community (and possibly functional) diversity.

## 3. Dietary Studies to Identify Niche Separation and Predict Climate Change Impacts on Polar Food Webs

Detailed insights into polar food webs are essential for predicting species and community resilience. Two articles in this Special Issue illustrate how DNA metabarcoding can resolve the dietary composition and trophic flexibility of polar organisms at an unprecedented taxonomic resolution. Masello et al. [15] found that two duck species sharing a coastal environment in the Falklands/Malvinas exploited different prey species, with only the flightless steamer duck consuming fish. Males and females of this species were also found to eat different fish species, suggesting that dietary niche segregation occurs at both the inter- and intraspecific level. Dischereit et al. [16] characterised the stomach contents of two co-occurring amphipods, *Themisto libellula* and *Themisto abyssorum*, which represent an important link between lower and higher tropic levels in the Arctic food web. They found that the prey composition of the two species was strongly differentiated during the Arctic summer, possibly reflecting segregation by depth. Moreover, *T. libellula* was found to consume a much broader range of prey organisms, including copepods and krill in Atlantic-influenced regions, and fish and ice-associated prey in polar regions, whereas the diet of *T. abyssorum* was consistently dominated by copepods. This greater trophic flexibility suggests that *T. libellula* may be better able to adapt to future changes in prey species abundance with the ongoing Atlantification of the Arctic Ocean.

## 4. Polar Adaptations

Another key focus of the polar genomics community is to understand the evolution of cold-adapted species and to identify local genetic adaptations. Cryonotothenoid fishes provide a classic example of cold adaptation in the Antarctic thanks to the innovation of antifreeze glycoproteins [17] and the loss of the classical inducible heat shock response [18]. Nevertheless, much remains unknown about the evolution and genome organisation of this group of Antarctic fishes. Cheng et al. [19] generated a chromosome-level genome assembly for the basal South American notothenoid, *Eleginops maclovinus*. They identified numerous chromosomal rearrangements as well as differences in the circadian gene repertoires of basal and derived notothenoids, thereby illustrating the utility of this resource as a reference for studying the evolution of circadian and other cold-adapted traits in the derived Antarctic clade. Winder et al. [20] provided insights into community-scale evolution by conducting a metagenomic survey of ice-binding proteins (IBPs) encoded by prokaryotic sea-ice and marine communities during the Arctic winter. Samples collected during the MOSAiC expedition (https://mosaic-expedition.org, accessed on 9 June 2023) were analysed to reveal structurally diverse IBPs encoded by microbial communities from interior ice and sea-ice interface habitats. These IBPs clustered taxonomically and exhibited diverse genomic contexts, suggesting that domain shuffling generates heterogeneous genetic architectures and functions, thereby adapting IBPs to specific habitats and lifestyles.

## 5. Technical Evaluations and Advancements

While technical advances are producing ever more robust insights into patterns of polar biodiversity, they can also sometimes bring into question inferences based on more classical approaches. For example, Becker and Pushkareva [21] compared the outcome of metabarcoding and metagenomic approaches applied to polar communities of soil bacteria. Using consistent bioinformatic workflows applied to the same samples, they found substantial differences in the inferred abundance of various taxa between the two methods, while metagenomics also identified a greater overall diversity of microbial taxa. These differences probably reflect the lower sequencing coverage and potential for PCR amplification bias inherent in the metabarcoding approach. Another cautionary tale is provided by Martínez et al. [22], who applied three different genetic markers (DNA barcoding, nuclear and mitochondrial SNPs) to assess phylogenetic relationships within the *Aequiyoldia eightsii* species complex. Incongruent results were obtained, with DNA barcoding revealing the presence of three distinct mitochondrial lineages within Antarctica, while the nuclear SNPs showed little in the way of differentiation. This appears to be due to a combination of heteroplasmy (a phenomenon linked to doubly uniparental inheritance in bivalves) and PCR amplification bias.

In summary, the articles included in this Special Issue showcase the research of a global community of researchers working on the genomics of polar organisms. These studies demonstrate how genomics techniques are increasingly being applied to polar species to answer important biological questions, particularly regarding the abilities of polar species to respond to our rapidly changing climate and how these abilities translate through to predictions of future ecosystem biodiversity. A major advantage of using genomics on polar species is the ability to derive robust biological insights, for example using whole genomes or metagenomes, from very limited samples (both in terms of numbers and volumes) gathered from extreme environments. This is especially important in the polar regions, where access for scientific research is severely restricted and there are limited possibilities to perform experiments. Finally, the articles in this Special Issue emphasise the diversity of contemporary polar genomic research, demonstrate the state of technological advancement in the field, and illustrate the potential of genomic approaches to advance our understanding of polar species, communities and ecosystems.
